# A Clinical Lecture

**Published:** 1888-05

**Authors:** D. A. K. Steele

**Affiliations:** Professor of Orthopedic Surgery in the College of Physicians and Surgeons; 1801 State Street


### A CLINICAL LECTURE.

BY D. A. K. STEELE, M. D.,

Professor ot Orthopedic Surgery in the College of Physicians and Surgeons.

[Reported by William Whitford.]

### DOUBLE CONGENITAL OBLIQUE INGUINAL HERNIA—MAC EWAN'S OPERATION.

Gentlemen: The next patient we present to-day will be this little child, with the following history: He is now 18 months of age; his mother states that soon after his birth she noticed a swelling in the lower part of the abdomen, just over the inguinal canal, the swelling appearing on either side simultaneously. Soon afterwards—when the child was some three weeks of age—the swelling was noticed extending lower down; along the course of the inguinal canal she observed that it increased when the child made any violent exertion, such as coughing, crying, straining, or any exercise that brought into play the diaphragm or abdominal muscles. Some weeks later she took the child to a dispensary where a truss was adjusted, but the instrument did not prevent the re-appearance of the swelling in the scrotum. During the past year a number of trusses have been applied, but none of them prevented the recurrence of the hernial swelling.

Now, when a little patient is brought to us suffering from a tumor of the scrotum it may mean one of a variety of diseases, and it is well for us to make a diagnosis by exclusion. Let us enumerate some of the various tumors that may occupy the scrotal pouch. We may in this locality have a tumor that may be either a hernia, hydrocele, orchitis, epididymitis, hæmatocele, varicocele, œdema, or a complication of two or more of these affections. How shall we differentiate these varied conditions? What will aid us in a correct diagnosis in a given case? For diagnostic or clinical purposes tumors of the scrotum may be divided into two general classes, to-wit: reducible and irreducible—reducible tumors including all herniæ (except strangulated), varicocele, and congenital hydroceles. Irreducible tumors of the scrotum include all tumors connected with the testicles, all hydroceles (except congenital), and strangulated hernia. Tumors of the tegumentary surface of the scrotum, such as inflammatory œdema, elephantiasis, and epithelioma, are usually so characteristic in their history as to offer no special impediments to a correct diagnosis.

Scrotal hernia may be mistaken for (1) hydrocele of tunica vaginalis, cord, or encysted hydrocele; (2) sarcocele of the testicle, simple, tuberculous, cystic, or malignant; (3) varicocele; (4) hæmatocele; (5) bubo or an undescended testis.

In scrotal hernia, as a rule, the tumor is soft and doughy to the touch, light weight, smooth and regular, painless unless inflamed or strangulated, of sudden advent from above downwards; resonant on percussion, fills the inguinal canal; has cough impulse, gurgles, is of normal color, opaque; may exisit on either side; the spermatic cord is concealed; does not fluctuate; aspiration is negative; bowels may be embarrassed. It can be reduced unless the hernia is strangulated or incarcerated.

In hydrocele of the testicle the tumor is ovoid or pyriform, develops slowly from below upwards; is firm, tense, and elastic; fluctuates, is transulucent, dull on percussion is irreducible; spermatic cord is neither concealed or displaced; the inguinal canal is empty; bowels unaffected; aspiration reveals fluid.

In congenital hydrocele the fluid disappears completely within the peritoneal cavity by compression of the tumor for a short time.

In sarcocele of the testicle the tumor is usually hard and resistant, heavy, often nodular and irregular; painful; grows slowly; dull or flat on percussion. The inguinal canal is empty; no impulse on coughing; bowels unaffected; irreducible; no auscultatory sounds. Simple sarcocle is chronic orchitis. Both the epididymis and body of the gland are affected. The cord is usually thickened. Abscess of the organ may occur. It is caused usually by injury followed by inflammatory deposits.

Tubercular sarcocele is met with most frequently in early manhood, and may occur in any constitution; in the strong and robust as well as the weak and cachectic; and although often associated with tuberculosis of other organs, it is common enough to find the tuberculous nidus in the epididymis, not as a sequence of gonorrhœal inflammation or some slight injury followed by inflammatory infiltration, as was formerly believed, but as a coincident. The progress is slow and insidious. The gland at first moderately enlarges with little or no pain, the hypertrophy being especially marked in the *globus major*. Presently the outline of the tumor becomes craggy or nodulated, and circles around the testicle from behind forwards in the form of a crescent. After several months, this adventitious tissue exceeds in size the testicle proper, and then it begins to soften at points and one or more abscesses burst and discharge a thin shreddy pus. The vas deferens is greatly enlarged.

In syphilitic sarcocele or gummata the history of the patient guides us in the diagnosis. Also, we find that the body of the gland is usually the seat of the infiltration which takes place in the connective tissue between the tubuli seminiferi, the epididymis undergoing little, if any, enlargement. The cord and vas deferens are unaffected. There is little or no tenderness, and the peculiar sensation elicited by squeezing a healthy testicle is absent. The tunica albuginea is very greatly thickened. Hydrocele is a frequent complication, and tapping is often required to establish a diagnosis.

Cystic tumors of the testis closely resemble hydrocele, and differ chiefly in being opaque instead of translucent. Aspiration should be practiced before pronouncing positivly upon their character.

Cancer of the testicle primarily invades the body of the gland, and almost invariably assumes the encephaloid form. Most observers doubt the existence of other varieties of malignant disease in this organ. The development of the disease is rapid. The patient has a sensation of weight, pain and dragging in the testis; the scrotum becomes distended, reddish or purplish, and the superficial veins are enlarged. The skin adheres to the gland, ulceration occurs, fungus protrudes, the inguinal glands are secondarily involved, and the patient by this time presents the characteristic cancerous cachexia.

In varicocele the tumor is knotty and irregular, like a bag of worms, bluish in color; most frequent upon the left side; increases in size upon the application of heat; developes gradually; is dull on percussion; fluctuation doubtful; spermatic cord not affected; inguinal canal not involved. No cough-impulse. It reduces spontaneously by any position that favors increased venous return, but returns immediately when the patient stands up notwithstanding pressure at the ring. There is weight and dragging in the scrotum.

In hæmatocele the advent is sudden, usually traumatic in origin; grows from below up if spontaneous; fluctuates until coagulation occurs; is at first soft, and hard after coagula form. It is pyriform in shape; ecchymotic, irreducible, heavy, dull on percussion. The spermatic cord is unaffected, and the inguinal canal empty.

There is often pallor and prostration from loss of blood. The bowels are unaffected.

Bubo is seldom mistaken for a scrotal tumor, and it is unnecessary to name differential points.

An undescended testicle is painful, and pressure upon it yields a peculiar sickening sensation. It is found wanting upon the side occupied by the tumor, and the scrotum is imperfectly developed upon the same side. It is sometimes mistaken for bubonocele.

Now, gentlemen, I trust the minute enumeration of diagnostic points mentioned in connection with these various scrotal tumors may not be considered tedious or unnecessary, and that a remembrance of them will aid you in making a correct diagnosis.

Let us refer to the points presented in the case of this little child. We have here a soft, elastic, compressible, reducible swelling, occupying both sides of the scrotal pouch; cough-impulse is present; gurgling is plainly felt. Upon flexing the thighs, elevating the hips, grasping the scrotum gently, by making moderate compression the tumor disappears within the abdominal cavity. Inversion of the bottom of the scrotum by the little finger enables us to readily follow the receeding mass through the dilated inguinal canal and distended inguinal ring into the abdominal cavity. With the history presented, and with the result of this physical examination, we are readily enabled to make a diagnosis of *double congenital oblique inguinal hernia.* Now, inasmuch as persistent efforts during the past year have been made by able surgeons to retain this hernia *in situ* by various trusses without success, what plan of treatment shall we devise? The treatment of this affection, as you know, may be divided into *palliative* and *radical*, or *curative.* Palliative treatment consists in the use of such apparatus, instruments, or trusses, fitting over the inguinal ring as will prevent the extrusion of the hernial tumor—fitted with sufficient nicety and adaptation to the contour of the parts as to inflict the minimum amount of pain, and yet with sufficient firmness to resist the intra-abdominal and intra-thoracic pressure induced by coughing, crying, straining during urination or defecation, or any exercise of the voluntary muscles that produce downward pressure upon the abdominal viscera. In a large proportion of cases a nicely adjusted truss, worn continuously during the first few months or year or two of infantile life, will suffice to effect a permanent occlusion of the distended inguinal ring, but occasionally we meet with cases, such as the one we have just examined, where a cure is impossible by means of any truss. Where it is impossible to retain the hernia within the abdomen, then it becomes necessary to resort to more radical measures—to a closure of the enlarged canal by means of a surgical operation. One of the best operations for this purpose is that devised by MacEwan, of Glasgow.

MacEwan's operation consists first in a thorough cleansing of the field of operation with soap and water, removing by means of turpentine all animal oil from the surface; the parts are then covered with a portion of lint, saturated with a bichloride solution. In the case of an adult the hair of the pubes and neighboring parts is closely shaven; the patient is then anæsthetized, the limb on the side of the swelling is flexed at the knee by a pillow just placed underneath. After having reduced the bowel, he makes an incision sufficient to expose the external abdominal ring. An exploration of the sac and its contents is then made, and the finger introduced through the canal examines abdominal aspects of the internal ring and the relative position of the epigastric artery. The operation may then be divided into two parts—the one relating to the establishment of a pad on the abdominal aspects of the internal ring; the other to the closure of the inguinal canal. The steps of the operation are as follows: He first frees and elevates the distal extremity of the sac, preserving along with it any adipose tissue that may be adherent to it. When that is done, he pulls upon the sac, and, while maintaining tension upon it, introduces the index finger into the inguinal canal, separating the sac from the cord, and from the parietes of the canal; then inserts the index finger outside the sac till it reaches the internal ring, and there separates with its tip the peritoneum for about half an inch around the abdominal aspects of the circumference of the ring.

Next, the stitch is secured firmly to the distal extremity of the sac, the end free is then passed in a proximal direction several times through the sac, so that when pulled upon the sac becomes folded upon itself like a curtain. The free end of this stitch, threaded on a hernial needle, is made to traverse the canal and to penetrate the anterior abdominal wall about an inch above the internal ring, the wound in the skin being pulled upward so as to allow the point of the needle to project through the abdominal muscles without penetrating the skin. The needle that he uses is cork-screw-shape with an eye in the point, being made rights and lefts. The thread is relieved from the extremity of the needle, when the latter is withdrawn; the thread is pulled through the abdominal wall, and when traction is made upon the sac, wrinkling upon itself, it is thrown into a series of folds, its distal extremity being drawn farthest backwards and upwards. An assistant maintains traction upon the stitch until the introduction of the sutures into the inguinal canal, and when this is completed the end of the stitch is secured by introducing its free extremity several times through the superficial layers of the external oblique muscle. The pad of peritoneum is then placed upon the abdominal side of the internal opening, where, owing to the abdominal aspect of the circumference of the internal ring having been refreshed, new adhesions may form. The sac baving been returned into the abdomen and secured to the abdominal circumference of the ring, the inguinal canal is closed outside of it in the following manner: The finger is introduced into the canal and lies between the inner and lower borders of the internal ring, the threaded hernia needle, then introduced and guided by the index finger, is made to penetrate the conjoined tendon in two places—*first*, from without inwards, near the lower border of the conjoined tendon; *second*, from within outwards as high as possible on the inner aspects of the canal. This double penetration of the conjoined tendon is accomplished by a single screw-like turn of the needle, one single thread is then withdrawn from the point of the needle by the index finger, and when this is accomplished, the needle along with the other extremity of the thread is removed. The inner side of the conjoined tendon is therefore penetrated twice by the thread, and a loop left on its abdominal aspect. Second, the other hernia needle, threaded with that portion of the stitch which comes from the lower border of the conjoined tendon, guided by the index finger into the inguinal canal, is introduced from within outwards through Poupart's ligament and the aponeurotic structures of the *transversalis internal and external oblique muscles;* it penetrates these structures at a point on a level with the lower stitch in the conjoined tendon. The needle is then completely freed from the thread and withdrawn. The needle is now threaded with the gut, which protrudes from the upper border of the conjoined tendon, and introduced from within outwards through the transversalis internal and external oblique muscles on a level corresponding with the upper stitch in the conjoined tendon. It is then quite free from the thread and withdrawn. There are now two thread ends on the outer surface of the external oblique muscle, and these are connected with a loop on the abdominal aspect of the conjoined tendon. To complete the suture the two thread-ends are drawn together and tied in a reef knot; this unites firmly the internal ring. The same stitch may be repeated lower down the canal, if thought desirable. In adults it is well to do .so. The pillars of the external ring are likewise brought together, and in order to avoid compression of the cord it ought to be examined before tightening each stitch; it ought to be freely movable. It is advisable to introduce all the necessary sutures before tightening any of them. When this is done, they may be all drawn, tied, and maintained so, while the operator's finger is introduced into the canal to ascertain the result. If satisfactory, they are then tied, beginning with the one at the internal ring, and taking up the others in order.

During the operation the skin is retracted from side to side to bring the parts into view and to enable the stitches to be fixed subcutaneously. When the retraction is relieved the skin falls into its normal position, the wound being opposite the external ring. Operation is therefore partly subcutaneous. When the canal has been brought together a decalcified bone drainage-tube is placed with its one extremity next to the external ring, the other projecting beyond the lower border of the external wound. A few chromatic gut-sutures are then introduced along the line of skin incision. Iodoform is dusted over the wound; a small portion of sublimated gauze is applied, and on top a sublimated pad, held in position by an aseptic bandage. As the patient is laid in bed, a pillow is placed under his knees, while his shoulders are slightly raised so as to relax the tissues about the canal. Temperature is taken night and morning, at the same time dressings are inspected and left undisturbed from fourteen to twenty-one days, unless they are stained or the temperature is abnormally high. From four to six weeks after the operation patient is allowed to rise from bed, but is not permitted to work until the end of the eighth week; is further advised not to lift heavy weights until the end of the third month. Adults engaged in laborious occupations are advised to wear a bandage and pad as a precautionary measure; and in the majority of children the closure is so complete and firm that further treatment by pad is unnecessary.

In the case of this little child, who is now under the influence of an anæsthetic, we will have to modify this operation, inasmuch as we have to deal with a congenital hernia in which the loop of bowel and testicle occupy a common sac; there is no tunica vaginalis proper. We will first make the preliminary incision thus; separate the sac from its connections; then open it transversely about an inch and a half above its distal extremity, and form the lower part into a tunica vaginalis by closing it with fine running catgut suture. The upper part of the sac is pulled down as far as possible now, split behind longitudinally, a portion of it closed around the cord, which is carefully preserved, and that portion which is left is now dealt with as the sac of an acquired hernia; passing a stitch through its distal portion through and through, so that it becomes folded up as a curtain, then with the finger I separate the attachments around the circumference of the abdominal ring. We then pass a needle through the abdominal wall an inch above the ring, bringing the thread out, where it is held by an assistant; drawing upon this cord forms the intra-abdominal pad or boss that forms a firm buttress to resist the impulse of the wave of intra-abdominal pressure.

We now proceed to close the internal ring in the manner described, passing two or three sutures along the course of the canal, drawing them snugly together, yet not tight enough to strangulate the cord. A drainage-tube is now passed from the upper angle of the wound and brought down through a button-hole in the bottom of the scrotum so as to afford perfect drainage. Parts are now irrigated with a 1-3000 solution of bichloride, the wound closed with interrupted catgut sutures, dusted lightly with iodoform, and hermetically sealed with cotton and collodion so as to prevent infection from the urine, which is liable to occur in a case of infants or young children. In closing up the wound in this way we reduce the risk of infection to a minimum. A heavy antiseptic dressing will now be applied, covered over with a starch bandage. The child will be kept as quiet as possible, small doses of camphorated tincture of opium administered from time to time, and the dressing will probably have to be changed every second day. The mother will be directed to keep the child lying on the left side as much as possible, so as to avoid soiling of the dressing during urination. The operation on the left side will be deferred until permanent healing of the right has taken place—say for two or three weeks.

A week later the child was presented to the class showing primary closure of the operation wound, except at the upper angle and the scrotal button-hole through which the drainage-tube emerged. Slight suppuration took place along the course of the drainage-tube. The highest temperature was 100.5° the day following the operation; the second day it dropped to 98½°, and remained normal subsequently, the child showing no symptoms or evidences of having undergone a serious or dangerous operation, the result so far being in every way eminently satisfactory.

1801 State Street.

